# Objective analysis of frailty-based 10-year outcomes of transcatheter and surgical aortic valve replacement

**DOI:** 10.1016/j.xjse.2025.100065

**Published:** 2025-07-31

**Authors:** Yoshitaka Naito, Arudo Hiraoka, Manami Himeno, Toshinobu Yoshida, Satoru Kishimoto, Genta Chikazawa, Yoshihito Okumura, Hidenori Yoshitaka

**Affiliations:** a Department of Rehabilitation, The Sakakibara Heart Institute of Okayama, Okayama, Japan; b Department of Cardiovascular Surgery, The Sakakibara Heart Institute of Okayama, Okayama, Japan; c Department of Cardiology, The Sakakibara Heart Institute of Okayama, Okayama, Japan; d Department of Pharmacy, The Sakakibara Heart Institute of Okayama, Okayama, Japan

**Keywords:** aortic stenosis, aortic valve replacement, transcatheter aortic valve implantation, frailty

## Abstract

**Objective:**

To compare the long-term outcomes of transcatheter aortic valve implantation (TAVI) and surgical aortic valve replacement (SAVR) in patients with aortic stenosis (AS) by adjusting not only for conventional risk scores but also for frailty, cognitive function, nutritional status, and comorbidities.

**Methods:**

This retrospective single-center study included 1258 patients (SAVR, n = 446; TAVI, n = 812) who underwent isolated elective procedures for AS between January 2014 and December 2024. Propensity score matching (1:1) was performed based on 19 baseline variables including age, Society of Thoracic Surgeons risk score, Clinical Frailty Scale, Mini-Mental State Examination, and Geriatric Nutritional Risk Index. The primary endpoint was a composite of all-cause mortality and cardiac readmission over a follow-up period of up to 10 years.

**Results:**

After matching, 114 patient pairs were obtained. The SAVR group had longer intensive care unit and hospital stays. However, there were no significant differences in 30-day mortality, stroke, pacemaker implantation, or readmission rates. Over long-term follow-up, a significantly better survival rate was seen in the SVAR group compared to the TAVI group (hazard ratio [HR], 0.16; 95% confidence interval [CI], 0.49-0.58; *P* = .005), while the readmission rate was similar in the 2 groups (HR, 1.0; 95% CI, 0.40-1.69; *P* = .93).

**Conclusions:**

After comprehensive adjustment for frailty, cognitive function, and nutritional status, SAVR demonstrated superior long-term survival compared to TAVI, with comparable readmission rates. Personalized treatment strategies for AS should incorporate broader patient-specific factors to guide optimal therapeutic decision making.

**Figure:**
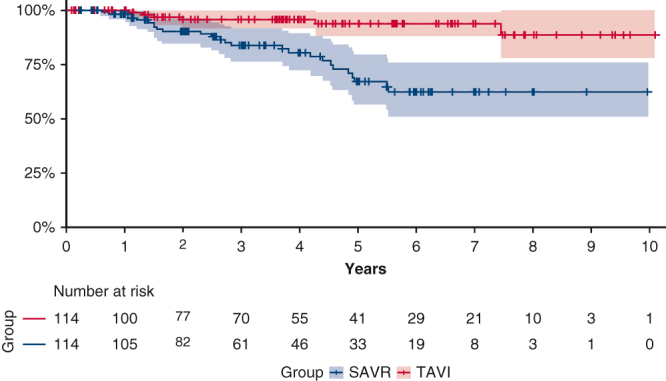
Kaplan-Meier curves for TAVI versus SAVR adjusted for patient background.

**Central Message**

After adjustment for patient background, mortality was significantly better for surgical aortic valve replacement compared to transcatheter aortic valve implantation.

**Perspective**

After adjusting for patient background, surgical aortic valve replacement showed superior long-term survival compared with transcatheter surgical aortic valve implantation; individualized treatment strategies for aortic stenosis should incorporate a wider range of patient background factors to guide optimal treatment decisions.

In recent years, transcatheter aortic valve implantation (TAVI) and surgical aortic valve replacement (SAVR) have emerged as interventional options for the management of patients with aortic stenosis (AS). Favorable outcomes of TAVI compared to medical management have been demonstrated,^1^ and several randomized controlled trials (RCTs) have shown noninferiority compared with SAVR in both high-risk and low-risk patients.^2^, ^3^, ^4^, ^5^, ^6^, ^7^ Consequently, TAVI is often preferred for patients deemed unsuitable for open-heart surgery or those at high surgical risk, with a growing interest in its application for low-risk patients as well. These findings remain a matter of debate, however. For example, the SAVR control group includes patients with previous open surgery, coronary artery disease, and concomitant surgeries, and thus conducting an objective comparison between pure isolated SAVR and TAVI groups is difficult. In addition, various factors, such as severe frailty, advanced dementia, and malnutrition, significantly influence mortality in TAVI patients,^8^, ^9^, ^10^, ^11^, ^12^, ^13^, ^14^, ^15^, ^16^, ^17^ but these factors are not incorporated into the Society of Thoracic Surgeons (STS) score.

Although risk stratification is typically performed using the STS score, the STS score may lack comprehensiveness in evaluating overall patient risk. Clinically, a significant proportion of elderly patients for whom TAVI is recommended present with frailty and other comorbidities.^9^^,^^18^^,^^19^ Thus, clinically optimal approaches should reflect frailty and comorbidities in addition to the STS score. Although many comparisons between TAVI and SAVR have been published, there remain few reports of comprehensively matched cohorts. The present study aimed to compare the long-term survival of recipients of isolated SAVR and TAVI by adjusting not only for STS score, but also for frailty, cognitive function, nutritional impairment, and comorbidities.

## Methods

### Study Population

**Figure 1:**
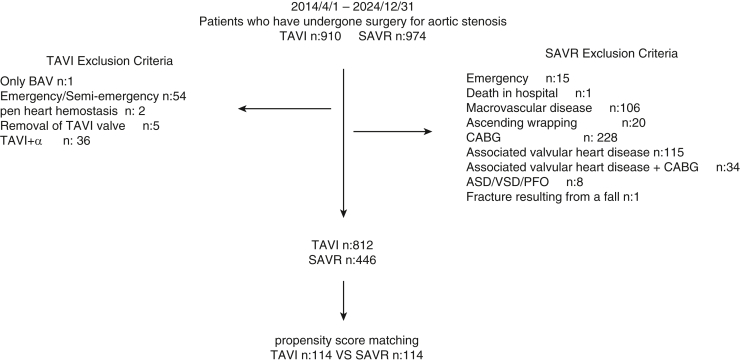
Flow chart of the study population. *TAVI*, Transcatheter aortic valve implantation; *SAVR*, surgical aortic valve replacement; *BAV*, bicuspid aortic valve; *CABG*, coronary artery bypass grafting; *ASD*, atrial septal defect; *VSD*, ventricular septal defect; *PFO*, patent foramen ovale.

This retrospective study at a single institution was approved by the institutional Ethics Committee in accordance with the ethical standards laid down in the 1964 Declaration of Helsinki (approval R202409-01; approved September 26, 2024). Written consent for use of their data was provided by all patients. A total of 974 patients who underwent SAVR and 910 patients who underwent TAVI for AS between January 2014 and December 2024 at the Sakakibara Heart Institute of Okayama were included in the study. Emergency and quasi-emergency cases, as well as cases involving combined major vascular and coronary surgeries, were excluded, resulting in a final analysis of 1258 cases (Figure 1). The decision to perform SAVR or TAVI was made following a comprehensive evaluation by the heart team.

### Physical, Cognitive Function, and Nutritional Assessment

The Clinical Frailty Scale (CFS) was used to assess physical frailty. The CFS provides an evaluation of daily living abilities and physical function, encompassing both basic and instrumental activities of daily living, mobility, activity level, and symptoms associated with disease. It is a comprehensive tool that produces a global score ranging from 1 (very healthy) to 9 (end-stage frailty). Cognitive function was assessed using the Mini-Mental State Examination (MMSE), which evaluates 6 cognitive domains: working memory, memory recall, orientation, attention, language, and visuospatial abilities. The MMSE has a maximum score of 30, with scores below 24 frequently used as a threshold for diagnosing dementia. These assessments were conducted preoperatively by rehabilitation staff.

The Geriatric Nutritional Risk Index (GNRI) was used to assess nutritional status. While various other indices, such as the Prognostic Nutritional Index and Controlling Nutritional Status, are available, GNRI is considered the most reflective of prognosis after TAVI. The GNRI formula is as follows: GNRI = 1.489 × albumin (g/L) + 41.7 × body weight (kg)/ideal body weight (kg). For this study, height and weight measured on the day before surgery were used, and albumin levels were obtained at hospital admission for the procedure.

### Clinical Follow-up

Patients were assessed during an outpatient visit at 1 month and 6 months after surgery and every year thereafter for a follow-up period of approximately 10 years after surgery. The primary endpoint was a composite of all-cause mortality and cardiac readmission owing to heart failure or arrythmia. Readmission for arrythmia was defined as a readmission for any arrhythmia requiring medical intervention. Heart failure was diagnosed based on clinical symptoms, physical signs, or objective evidence of pulmonary congestion.

### Statistical Analysis

Continuous data are presented as a mean ± standard deviation and were analyzed using the 2-tailed *t* test or compared with a Mann-Whitney *U* test for independent data, as appropriate. Categorical variables, given as count and percentage of patients, were compared using the χ^2^ test or Fisher exact test. Survival rates between groups were compared using the Kaplan-Meier model and the log-rank test. (The survival curve was cut off when the observer counted fewer than 10 cases.) When comparing readmissions, mortality was treated as a competing event that inhibited the occurrence of readmission. Cumulative incidence functions that took competing risks into account were estimated using the Fine and Gray model. Mortality was compared based on all-cause mortality. A *P* value < .05 was considered significant. Data analysis was performed using SPSS version 18.0 and R. Propensity score matching was performed to reduce the risk of bias and potential confounding factors in treatment selection. 1:1 closest matching without replacement was performed on the logit of the propensity score, with a caliper value of 0.25. Adjustment variables were selected based on factors showing differences between groups and findings from previous studies.

**Figure E1:**
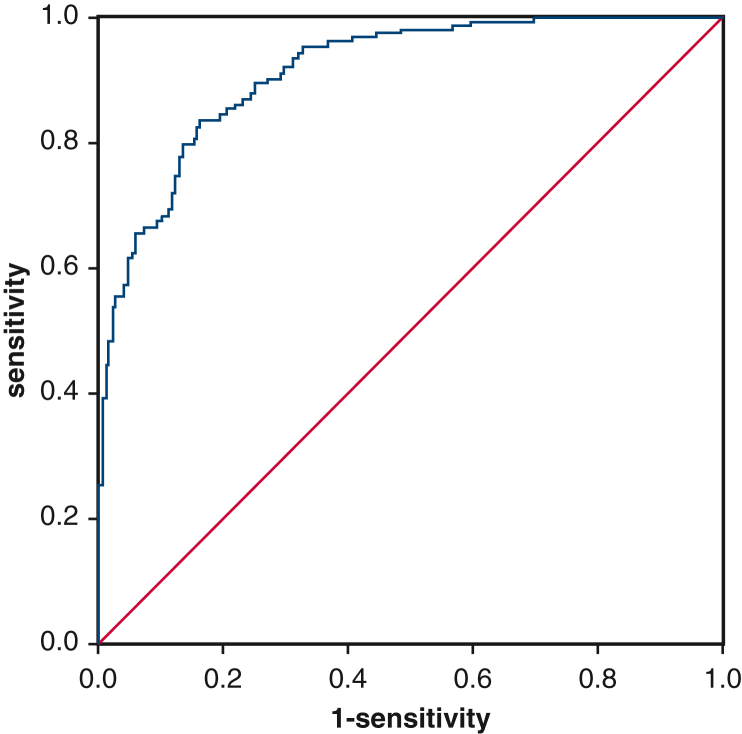
The matching model achieved good discriminatory power (C-statistic: 0.90).

The propensity score was obtained using a multivariate logistic regression model with SAVR versus TAVI as the variables and 19 baseline characteristics: age, sex, hypertension, diabetes mellitus, atrial fibrillation, STS score, hemoglobin, GNRI, creatinine, B-type natriuretic peptide, left ventricular ejection fraction, mean pressure gradient, aortic valve velocity, aortic valve area, mitral regurgitation, tricuspid regurgitation, tricuspid regurgitation pressure gradient, CFS, and MMSE. The matching model was evaluated using the C-statistic, an index of agreement reflecting the area under the receiver operating characteristic curve, from the logistic regression model used to estimate the propensity score. The C-statistic was 0.90, indicating excellent discrimination ability between the matched groups (Figure E1).

## Results

### Unmatched and Matched Comparisons of Patient Demographics

Among the 1884 patients who underwent elective surgery, 1258 (SAVR, n = 446; TAVI, n = 812) were included in the analysis (Figure 1). The overall mean age was 81 ± 8 years, and the cohort was 63% being female. At baseline, patients in the TAVI group were generally older (mean age, 74 ± 8 years vs 85 ± 4 years; *P* = .001), more likely to be female (56% vs 67%; *P* = .001), had higher STS scores (mean, 8 ± 6 vs 11 ± 7; *P* = .001), and the percentage of hypertension (71% vs 82%; *P* = .001), chronic kidney disease (17% vs 25%; *P* = .003), and atrial fibrillation (6% vs 19%; *P* = .001) were greater in the TAVI group.

**Figure 2:**
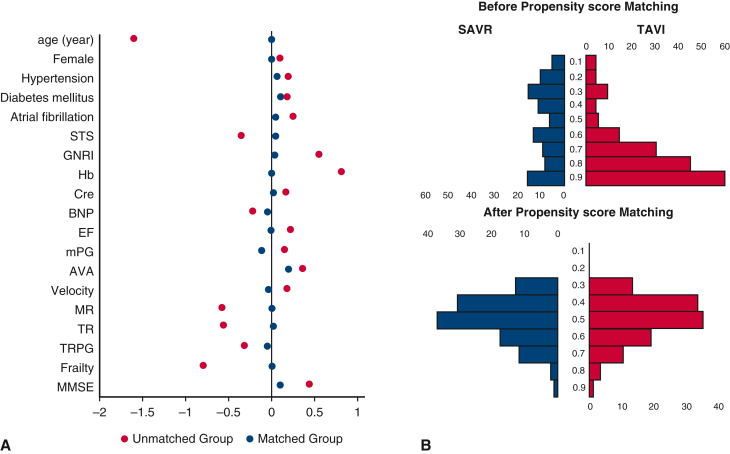
Standardized mean difference and distribution of propensity scores before and after matching. A, Standardized mean difference before and after propensity score matching. B, Distribution of propensity scores before and after matching. *SAVR*, Surgical aortic valve replacement; *TAVI*, transcatheter aortic valve implantation; *STS*, Society of Thoracic Surgeons; *GNRI*, Geriatric Nutritional Risk Index; *Hb*, hemoglobin; *Cre*, creatinine; *BNP*, brain natriuretic peptide; *EF*, ejection fraction; mPG, mean pressure gradient; *AVA*, aortic valve area; *MR*, mitral regurgitation; *TR*, tricuspid regurgitation; *TRPG*, tricuspid regurgitation pressure gradient; *Frailty*, Clinical Frailty Scale; *MMSE*, Mini-Mental State Examination.

After propensity score matching, 114 pairs were selected from each group (SAVR and TAVI). As presented in Table 1, no significant differences in preoperative characteristics were observed between the matched groups. Significant imbalance of preoperative characteristics was adjusted well, as shown in Figures 2, *A* and *B*. The matched characteristics included equivalent age (mean, 81 ± 4 years vs 81 ± 4 years; *P* = .98), STS score (11 ± 7 vs 11 ± 7; *P* = .66), proportion of females (60% vs 61%; *P* = .89), hypertension (74% vs 79%; *P* = .43), diabetes mellitus (35% vs 36%; *P* = .89), and atrial fibrillation (7% vs 10%; *P* = .66).

**Table 1:** Unmatched and matched comparisons of baseline demographics

Characteristic	Before propensity score matching				After propensity score matching			
	SAVR (N = 446)	TAVI (N = 812)	*P* value	SMD	SAVR (N = 114)	TAVI (N = 114)	*P* value	SMD
Age, y, mean ± SD	74 ± 8	85 ± 4	.001	1.519	81 ± 4	81 ± 4	.98	0.004
Age ≥60 y, n (%)	32 (7)	0	.001	0.393	-	-	-	
Age 61-70 y, n (%)	86 (19)	5 (1)	.001	0.656	1 (1)	2 (2)	1.00	0.01
Age 71-80 y, n (%)	214 (48)	110 (14)	.001	0.804	49 (43)	38 (33)	.17	0.077
Age 81-90 y, n (%)	111 (25)	594 (73)	.001	1.102	62 (54)	72 (63)	.22	0.100
Age ≥91 y, n (%)	3 (1)	103 (13)	.001	0.496	2 (2)	2 (2)	1.00	0.179
Female sex, n (%)	251 (56)	547 (67)	.001	0.230	68 (60)	70 (61)	.89	0.036
BMI, mean ± SD	23 ± 3	22 ± 3	.001	0.298	22 ± 3	22 ± 3	.51	0.067
Hypertension, n (%)	315 (71)	664 (82)	.001	0.264	85 (74)	91 (79)	.43	0.126
Dyslipidemia, n (%)	232 (52)	375 (46)	.039	0.124	62 (54)	6% (57)	.79	0.053
Diabetes mellitus, n (%)	156 (35)	278 (34)	.80	0.016	40 (35)	42 (36)	.89	0.037
Chronic kidney disease, n (%)	78 (17)	202 (25)	.003	0.182	22 (1)9	21 (18)	1.00	0.022
Orthopedic, n (%)	99 (22)	162 (20)	.34	0.055	26 (23)	27 (24)	1.00	0.021
Cerebrovascular incidence, n (%)	115 (26)	219 (27)	.68	0.026	34 (30)	32 (28)	.88	0.023
Peripheral artery disease, n (%)	17 (4)	39 (5)	.47	0.049	5 (4)	6 (5)	1.00	0.041
Atrial fibrillation, n (%)	26 (6)	154 (19)	.001	0.405	8 (7)	11 (10)	.63	0.095
STS score, mean ± SD	8 ± 6	11 ± 7	.001	−0.350	11 ± 7	11 ± 7	.66	0.050
Hemoglobin, g/dL, mean ± SD	13 ± 1	11 ± 1	.001	0.820	11 ± 1	11 ± 1	.88	0.019
Albumin, g/dL, mean ± SD	3.9 ± 0.4	3.6 ± 0.4	.001	0.787	3.8 ± 0.4	3.8 ± 0.4	.88	0.020
Creatinine, mg/dL, mean ± SD	1.6 ± 2	1.2 ± 1	.001	0.165	1.4 ± 2	1.3 ± 2	.88	0.020
BNP, g/mL, mean ± SD	223 ± 448	316 ± 429	.001	−0.220	259 ± 421	279 ± 364	.70	−0.050
ACE/ARB, n (%)	230 (52)	398 (49)	.41	0.050	53 (46)	55 (48)	.47	0.053
SGLT2, n (%)	17 (4)	37 (5)	.56	0.037	4 (4)	5 (4)	1.00	0.045
ARNI, n (%)	5 (1)	11 (1)	.79	0.021	1 (1)	0	1.00	0.13
Statin, n (%)	221 (50)	347 (43)	.024	0.13	47 (41)	59 (52)	.14	0.21
Antiplatelet, n (%)	108 (24)	233 (29)	.09	0.12	30 (26)	33 (29)	.12	0.05
Anticoagulant, n (%)	53 (12)	136 (17)	.021	0.14	18 (16)	11 (10)	.23	0.18
Diuretic, n (%)	81 (18)	315 (39)	.001	0.47	30 (26)	33 (29)	.76	0.05
MRA, n (%)	43 (10)	139 (17)	.001	0.22	16 (14)	14 (12)	.84	0.045
β-blocker, n (%)	116 (26)	254 (31)	.052	0.11	24 (21)	31 (27)	.35	0.14

*SAVR*, Surgical aortic valve replacement; *TAVI*, transcatheter aortic valve implantation; *SMD*, standardized mean difference; *BMI*, body mass index; *STS*, Society of Thoracic Surgeons; *BNP*, brain natriuretic peptide; *ACE*, angiotensin-converting enzyme; *ARB*, angiotensin II receptor blocker; *SGLT2*, sodium–glucose cotransporter 2; *ARNI*, angiotensin receptor–neprilysin inhibitor; *MRA*, mineralocorticoid receptor antagonist.

### Unmatched and Matched Comparisons of Echocardiographic Parameters

Echocardiography showed significant differences in the severity of AS, cardiac function, and comorbid valvular disease before matching, but there were no significant differences in the severity of AS after matching, including mean pressure gradient (51 ± 17 mm Hg vs 53 ± 17 mm Hg; *P* = .34), peak velocity (4.8 ± 0.7 m/s vs 4.8 ± 0.7 m/s; *P* = .72), and aortic valve area (0.7 ± 0.2 cm^2^ vs 0.7 ± 0.2 cm^2^; *P* = .82). Similarly, no differences were observed in parameters of cardiac function, including left ventricular ejection fraction (mean, 62 ± 10% vs 62 ± 10%; *P* = .94), SVI (mean, 49 ± 10 mL/m^2^ vs 50 ± 11 mL/m^2^; *P* = .85), valve status, mitral regurgitation grade (mean, 1.8 ± 0.6 vs 1.8 ± 0.6; *P* = 1.00) and tricuspid regurgitation grade (1.7 ± 0.6 vs 1.7 ± 0.6; *P* = .84) (Table 2).

**Table 2:** Unmatched and matched comparisons of baseline echocardiographic data

Variable	Before propensity score matching				After propensity score matching			
	SAVR (N = 446), mean ± SD	TAVI (N = 812), mean ± SD	*P* value	SMD	SAVR (N = 114), mean ± SD	TAVI (N = 114), mean ± SD	*P* value	SMD
Ejection fraction, %	64 ± 10	61 ± 11	.001	0.234	62 ± 10	62 ± 10	.94	−0.010
Stroke volume index, mL	50 ± 12	48 ± 13	.001	0.150	49 ± 10	50 ± 11	.85	0.024
AV gradient, mm Hg	51 ± 17	48 ± 16	.001	0.167	51 ± 17	53 ± 17	.34	−0.125
AV velocity, m/s	4.7 ± 0.7	4.5 ± 0.7	.001	0.188	4.8 ± 0.7	4.8 ± 0.7	.72	−0.046
AVA, cm^2^	0.74 ± 0.2	0.66 ± 0.1	.001	0.341	0.7 ± 0.2	0.7 ± 0.2	.82	0.263
AR grade	1.9 ± 1	2 ± 0.7	.13	0.031	2 ± 0.9	2 ± 0.8	.12	0.001
MR grade	1.6 ± 0.6	1.9 ± 0.7	.001	−0.570	1.8 ± 0.6	1.8 ± 0.6	1.00	0.001
TR grade	1.4 ± 0.7	1.8 ± 0.7	.001	−0.561	1.7 ± 0.6	1.7 ± 0.6	.84	0.002
TRPG, mm Hg	24 ± 7	27 ± 9	.001	0.312	26 ± 6	26 ± 9	.67	−0.051

*SAVR*, Surgical aortic valve replacement; *TAVI*, transcatheter aortic valve implantation; *SMD*, standardized mean difference; *AV*, aortic valve; *AVA*, aortic valve area; *AR*, aortic regurgitation; *MR*, mitral regurgitation; *TR*, tricuspid regurgitation; *TRPG*, tricuspid regurgitation pressure gradient.

### Unmatched and Matched Comparisons of Frailty, Cognitive Status, and Nutritional Status

Before propensity score matching, there were significant differences in frailty, cognitive, and nutrition status between the SAVR and TAVI groups. After matching, there were no significant differences in frailty (mean CFS score, 4 ± 1 vs 4 ± 1; *P* = .47), cognitive function (mean MMSE, 26 ± 2 vs 26 ± 4; *P* = .47), and nutritional status (mean GNRI, 100 ± 10 vs 100 ± 9; *P* = .81) (Table 3).

**Table 3:** Unmatched and matched comparisons of physical, cognitive function, and nutrition data

Variable	Before propensity score matching				After propensity score matching			
	SAVR (N = 446)	TAVI (N = 812)	*P* value	SMD	SAVR (N = 114)	TAVI (N = 114)	*P* value	SMD
NYHA class Ⅲ-Ⅳ, n (%)	65 (15)	187 (23)	.001	0.392	14 (12)	21 (18)	.27	0.171
CFS, mean ± SD	3.4 ± 1	4.4 ± 1	.001	−0.790	4 ± 1	4 ± 1	.47	0.001
CFS, n (%)								
1	13 (3)	16 (2)	.32	0.061	4 (4)	3 (3)	1.00	0.051
2	75 (17)	40 (5)	.001	0.389	11 (10)	15 (13)	.53	0.110
3	131 (29)	91 (11)	.001	0.464	25 (22)	23 (20)	.87	0.043
4	156 (35)	278 (34)	.80	0.016	45 (40)	43 (38)	.89	0.036
5	59 (13)	236 (29)	.001	0.395	23 (20)	24 (21)	1.00	0.022
6	7 (2)	123 (15)	.001	0.506	3 (3)	2 (2)	1.00	0.060
7	5 (1)	28 (3)	.015	0.156	3 (3)	4 (4)	1.00	0.051
MMSE, mean ± SD	26 ± 3	24 ± 5	.001	0.445	26 ± 2	26 ± 4	.47	0.111
GNRI, mean ± SD	103 ± 12	96 ± 12	.001	0.551	100 ± 10	100 ± 9	.81	0.032

*SAVR*, Surgical aortic valve replacement; *TAVI*, transcatheter aortic valve implantation; *SMD*, standardized mean difference; *NYHA*, New York Heart Association; *CFS*, Clinical Frailty Scale; *MMSE*, Mini-Mental Status Examination; *GNRI*, Geriatric Nutritional Risk Index.

### Unmatched and Matched Comparisons of Early and Long-Term Outcomes

Unmatched and matched comparisons of surgical data are shown in Table E1. Perioperative and 30-day outcomes before and after matching are shown in Table E2. The SAVR group had longer intensive care unit and hospital lengths of stay compared with the TAVI group before and after matching. Although the incidence of pacemaker implantation and stroke were significantly higher in the TAVI group before matching, there were no significant differences in the incidences of pacemaker implantation, stroke, rehospitalization, and death between the groups after matching.

**Figure 3:**
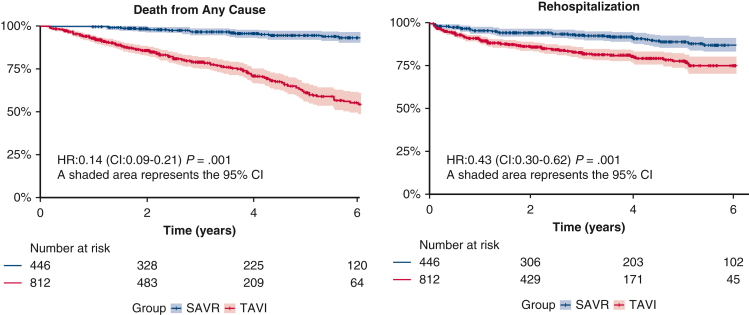
Kaplan-Meier survival curves for rehospitalization and all-cause mortality before matching. *Shaded areas* indicate 95% confidence intervals (CI). *TAVI*, Transcatheter aortic valve implantation; *SAVR*, surgical aortic valve replacement; *HR*, hazard ratio.

**Figure 4:**
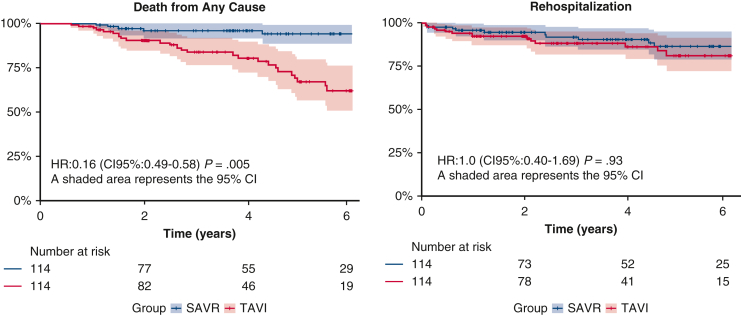
Kaplan-Meier survival curves for rehospitalization and all-cause mortality after matching. *Shaded areas* indicate 95% confidence intervals (CIs). *TAVI*, Transcatheter aortic valve implantation; *SAVR*, surgical aortic valve replacement; *HR*, hazard ratio.

The observation period extended up to approximately 10 years, and details of mortality are provided in Tables E3 and E4. A total of 232 patients (18%) died during the observation period. The survival rate was significantly better in the SAVR group compared to the TAVI group, with a hazard ratio (HR) of 0.14 (95% confidence interval [CI], 0.09-0.21; *P* = .001; median not reached in SAVR and 6.4 years in TAVI) (Figure 3). Similarly, the readmission rate was lower in the SAVR group (HR, 0.43; 95% CI, 0.30-0.62; *P* = .001). After matching, death occurred in 33 patients (14%) during follow-up. The mortality rate was significantly better in the SAVR group, with an HR of 0.16 (95% CI, 0.49-0.58; *P* = .005; median not reached for both SAVR and TAVI). However, there was no significant difference in readmission rate between the 2 groups (HR, 1.0; 95% CI, 0.40-1.69; *P* = .93) (Figure 4).

**Figure E2:**
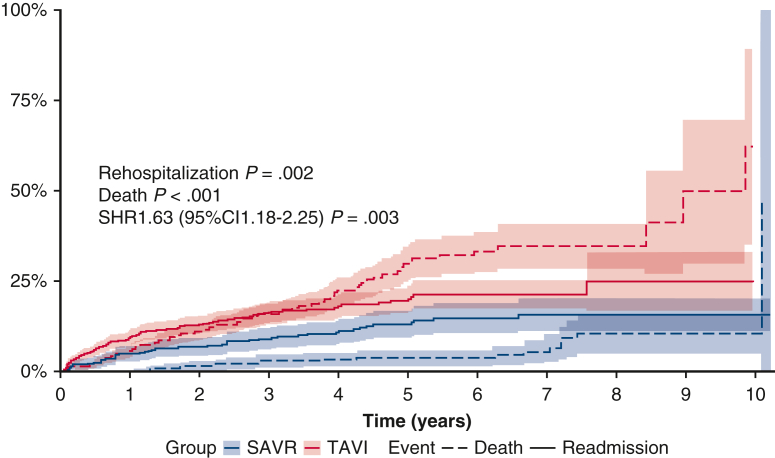
Cumulative incidence functions before propensity score matching using the Gray test. *TAVI*, Transcatheter aortic valve implantation; *SAVR*, surgical aortic valve replacement; *sHR*, subdistribution hazard ratio; *CI*, confidence interval.

When evaluating readmission, cumulative incidence function (CIF) analysis using Gray's test was performed, because death as a competing risk may affect readmission. Before propensity score matching, the cumulative incidence of readmission was significantly higher than in the TAVI group compared to the SAVR group (*P* < .001). The median time to readmission was not reached in the SAVR group and was 9.8 years in the TAVI group. The subdistribution HR by the Gray model was 1.63 (95% CI, 1.18-2.25; *P* < .001), indicating that the cumulative incidence of readmission in the TAVI group was significantly higher even when considering death as a competing risk (Figure E2).

**Figure E3:**
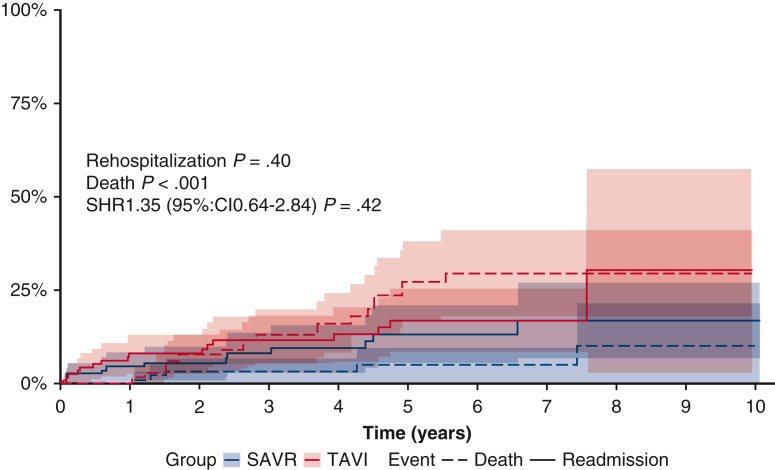
Cumulative incidence functions after propensity score matching using the Gray test. *TAVI*, Transcatheter aortic valve implantation; *SAVR*, surgical aortic valve replacement; *sHR*, subdistribution hazard ratio; *CI*, confidence interval.

After matching, the difference in cumulative readmission rate between the groups was no longer statistically significant (Gray test, *P* = .408; subdistribution HR, 1.35; 95% CI, 0.64-2.84; *P* = .42). In contrast, the cumulative mortality rate remained significantly higher in the TAVI group (Gray test, *P* < .001) (Figure E3).

## Discussion

The main results of this study can be summarized as follows: (1) in unmatched comparisons, long-term outcomes in terms of both mortality and rehospitalization rates were better in the SAVR group compared to the TAVI group; (2) after propensity score matching based on cognitive function and frailty status, perioperative intensive care unit and hospital lengths of stay were significantly longer in the SAVR group, but there were no significant between-group differences in 30-day mortality, rehospitalization, and stroke incidence; and (3) long-term survival was significantly better in the SAVR group, while the rehospitalization rate was equivalent in the 2 groups.

Good early outcomes of TAVI has been reported in several studies, and recently published guidelines recommend TAVI for moderate-risk patients age ≥75 years in Europe and for patients age ≥65 years in the United States.^20^^,^^21^ As a result, TAVI is becoming more common among younger patients, and it is estimated that approximately one-half of patients age ≤65 years with isolated AS have undergone TAVI in the United States.^22^ Previous reports show a lower incidence of prosthetic–patient mismatch and hemodynamic valve dysfunction and higher rates of paravalvular leak and pacemaker implantation in TAVI recipients.^23^^,^^24^

SAVR and TAVI each carry a risk of complications, which must be taken into consideration when selecting the optimal treatment. Additionally, patients with a history of previous cardiac surgery and concomitant surgery were included in previous RCTs for SAVR versus TAVI.^25^ Although noninferiority was shown in these RCTs, 5-year outcomes were insignificantly better in the SAVR group compared to TAVI with a balloon expandable stent.^3^ Therefore, a joint statement from the European Association for Cardio-Thoracic Surgery and STS for AVR in low-risk patients required results of isolated SAVR and TAVI cohort for more objective comparison. Furthermore, cognitive function and frailty status were not included when creating the RCT cohort.

In this study, previously unaccounted for background factors, including frailty, cognitive function, and nutritional status, were meticulously adjusted for in a propensity score–matched cohort of SAVR and TAVI patients to facilitate a more objective comparison. The findings show that the sole advantage of TAVI was a reduced length of hospital stay. However, recent studies have reported a high incidence of hospital-associated disorders among TAVI patients, which adversely impacted prognoses (24.4% post-TAVI vs 5.2% following open-heart surgery).^26^^,^^27^ A shorter hospitalization period may shorten rehabilitation time, thereby impeding the patient's capacity to enhance their functional activity. Therefore, interactions that prevent decline during hospitalization are necessary, and because the length of hospital length of stay is short, continuous support is needed to maintain activity levels after discharge.

From a prognostic perspective, the SAVR group demonstrated superior long-term outcomes. In their meta-analysis of RCTs, Braili and colleagues^28^ reported that while TAVI exhibited a higher survival rate within the first 6 months, SAVR was associated with better survival beyond just 1 year. Similarly, Takagi and colleagues^29^ analyzed mortality rates over a follow-up period exceeding 5 years, with findings that further reinforced the survival advantage of SAVR. There is a growing body of evidence supporting the superiority of SAVR in terms of long-term survival. Post-TAVI treatment strategies include valve-in-valve implantation and SAVR with TAVI valve explantation. Comparative studies of SAVR and valve-in-valve implantation demonstrated superior long-term survival rates in the SAVR cohort.^30^ Additionally, SAVR with TAVI valve explantation was associated with increased mortality owing to the complexity of additional aortic surgery.^31^^,^^32^ When managing younger or low-risk patients with a longer life expectancy, the potential need for future reintervention must be carefully considered. For these reasons, formulating an optimal long-term treatment strategy requires a comprehensive evaluation of individual factors, including age, anatomic characteristics, comorbid conditions, frailty, cognitive function, nutritional status, and the patient's personal preferences. Treatment decisions should not be dictated solely by age or risk stratification but should be tailored to the patient's overall clinical profile. Multidisciplinary cardiac teams at each institution must consistently adopt a flexible, patient-centered approach in selecting the most appropriate therapeutic intervention.

### Study Limitations

This study has several limitations. First, the study was not a prospective randomized analysis, and thus TAVI was generally selected after careful consideration. This selection bias might not have been completely adjusted for by propensity score matching, even though the selection of surgical procedure could not be decided randomly and prospectively when the best approach was sought for each patient. Furthermore, the matching variables were selected based on previous studies but failed to take into account unmeasured confounding factors. Second, differences in the procedures and devices used in TAVI and SAVR may have influenced the treatment outcomes. In particular, the use rate of balloon-expandable valves was high in the TAVI group (approximately 70%), and bias due to the device may have influenced the interpretation of the results. Third, Japan is one of the most rapidly aging countries in the world, as reflected in the study population, which included a large number of patients with high STS scores. The STS in this cohort was relatively high compared to recent studies. Finally, the various changes in the indications for TAVI and the learning curve and development of devices over the past decade may hinder evaluation of the results.

## Conclusions

After adjustments based on unaccounted background factors, including frailty, cognitive function, and nutritional status, SAVR had a better long-term survival rate than TAVI, with a similar readmission rate. A customized treatment strategy for each patient requires a comprehensive evaluation of age, anatomy, frailty, and other patient-specific factors.

## Conflict of Interest Statement

The authors reported no conflicts of interest.

The *Journal* policy requires editors and reviewers to disclose conflicts of interest and to decline handling or reviewing manuscripts for which they may have a conflict of interest. The editors and reviewers of this article have no conflicts of interest.

## Figures and Tables

**Table E1:** Unmatched and matched comparisons of surgical data

Variable	Before propensity score matching				After propensity score matching			
	SAVR (N = 446)	TAVI (N = 812)	*P* value	SMD	SAVR (N = 114)	TAVI (M = 114)	*P* value	SMD
Operation time, min, mean ± SD	248 ± 52	80 ± 28	.001	3.971	240 ± 54	83 ± 35	.001	3.433
Transfemoral, n (%)	-	722 (89)	-		-	96 (84)	-	
Transapical, n (%)	-	41 (5)	-		-	8 (7)	-	
Trans-subclavian, n (%)	-	48 (6)	-		-	9 (8)	-	
Direct aortic, n (%)	-	1 (0.1)	-		-	1 (1)	-	
Sapien XT, n (%)	-	74 (9)	-		-	16 (14)	-	
Sapien 3, n (%)	-	397 (49)	-		-	56 (49)	-	
Sapien 3 UR, n (%)	-	31 (4)	-		-	5 (4)	-	
Core valve, n (%)	-	29 (4)	-		-	6 (5)	-	
Evolute R, n (%)	-	77 (9)	-		-	17 (15)	-	
Evolute Pro, n (%)	-	58 (7)	-		-	3 (3)	-	
Evolute Pro+, n (%)	-	94 (12)	-		-	8 (7)	-	
Evolute FX, n (%)	-	18 (2)	-		-	-	-	
Navitor, n (%)	-	34 (4)	-		-	3 (3)	-	
CEP Magna EASE, n (%)	306 (69)		-		80 (70)		-	
Trifecta, n (%)	7 (2)		-		2 (2)		-	
Mosaic, n (%)	7 (2)		-		4 (4)		-	
Mirtoflow, n (%)	2 (0)		-		1 (1)		-	
Crown PRT, n (%)	4 (1)		-		1 (1)		-	
Inspiris Resilia, n (%)	10 (2)		-				-	
Avalus, n (%)	15 (3)		-		3 (3)		-	
Intuity Elite, n (%)	12 (3)		-		5 (4)		-	
Percival, n (%)	46 (10)		-		18 (16)		-	
ATS AP360, n (%)	31 (7)		-				-	
ATS standard, n (%)	1 (0)		-				-	
SJM regent, n (%)	4 (1)		-				-	
SJM standard, n (%)	1 (0)		-				-	

*SAVR*, Surgical aortic valve replacement; *TAVI*, transcatheter aortic valve implantation; *SMD*, standardized mean difference.

**Table E2:** Unmatched and matched comparison of periprocedural and discharge medications and 30-day outcomes

Variable	Before propensity score matching				After propensity score matching			
	Surgery (n = 446)	TAVI (N = 812)	*P* value	SMD	Surgery (N = 114)	TAVI (N = 114)	*P* value	SMD
ICU LOS, d, mean SD	3 ± 2	2 ± 1	.001	0.889	3 ± 2	2 ± 1	.001	0.830
Hospital LOS, d, mean SD	22 ± 9	11 ± 8	.001	1.181	23 ± 11	9 ± 7	.003	1.479
New permanent pacemaker, n (%)	12 (3)	52 (6)	.004	0.179	5 (4)	4 (4)	1.00	0.212
All-cause death, n (%)	0	1 (0.1)	1.00	0.050	0	0	-	-
Stroke, n (%)	3 (1)	24 (3)	.007	0.172	0	3 (2)	.24	0.232
Rehospitalization, n (%)	4 (1)	10 (1)	.40	0.033	1 (1)	1 (1)	1.00	0.001
ACE/ARB, n (%)	133 (30)	324 (40)	.001	0.21	35 (31)	51 (45)	.05	0.29
SGLT2, n (%)	34 (8)	38 (5)	.042	0.12	7 (6)	6 (5)	1.00	0.038
ARNI, n (%)	3 (1)	16 (2)	.09	0.11	1 (1)	0	1.00	0.13
Statin, n (%)	223 (50)	326 (40)	.001	0.13	50 (44)	56 (49)	.50	0.10
Antiplatelet, n (%)	380 (85)	701 (86)	.55	0.035	25 (22)	6 (5)	.001	0.50
Anticoagulant, n (%)	434 (97)	184 (23)	.001	2.23	110 (96)	21 (18)	.001	2.25
Diuretic, n (%)	305 (68)	518 (64)	.12	0.09	82 (72)	62 (54)	.009	0.37
MRA, n (%)	124 (28)	178 (22)	.023	0.13	32 (28)	20 (18)	.08	0.25
β-blocker, n (%)	396 (89)	358 (44)	.001	1.03	98 (86)	49 (43)	.001	1.00

*TAVI*, Transcatheter aortic valve implantation; *SMD*, standardized mean difference; *ICU*, intensive care unit; *LOS*, length of stay; *ACE*, angiotensin-converting enzyme; *ARB*, angiotensin II receptor blocker; *SGLT2*, sodium–glucose cotransporter 2; *ARNI*, angiotensin receptor–neprilysin inhibitor; *MRA*, mineralocorticoid receptor antagonist.

**Table E3:** Causes of death, unmatched and matched comparisons

Cause of death	Before propensity score matching, n (%)		After propensity score matching, n (%)	
	SAVR (N = 28)	TAVI (N = 204)	SAVR (N = 7)	TAVI (N = 26)
Cardiac disease∗	4 (14)	57 (28)	1 (14)	9 (35)
Infection/sepsis†	3 (11)	38 (19)	1 (14)	9 (35)
Cancer	9 (32)	24 (12)	2 (29)	3 (12)
Renal failure	0	14 (7)	-	-
Stroke	3 (11)	11 (5)	1 (14)	2 (8)
Senility	0	10 (5)	-	-
Gastrointestinal‡	0	3 (1)	-	-
Leukemia	0	2 (1)	-	-
Unknown (including sudden death)	9 (32)	45 (22)	2 (29)	3 (12)

*SAVR*, Surgical aortic valve replacement; *TAVI*, transcatheter aortic valve implantation.

∗ Cardiac disease: heart failure, arrhythmia, myocardial infarction, artificial valve infection, etc.

† Infection: pneumonia, aspiration pneumonia, etc.

‡ Gastrointestinal: cholecystitis, gastrointestinal hemorrhage, etc.

**Table E4:** Unmatched and matched comparisons of details of heart disease deaths

Cause of death	Before propensity score matching, n (%)		After propensity score matching, n (%)	
	SAVR (N = 4)	TAVI (N = 57)	SAVR (N = 1)	TAVI (N = 9)
Heart failure	1 (25)	42 (74)	1 (100)	6 (67)
Myocardial infarction	1 (25)	2 (4)	-	-
Arrhythmia	1 (25)	4 (7)	-	-
Infectious endocarditis	0	3 (5)	-	-
Aortic aneurysm rupture	0	5 (9)	0	3 (33)
Peripheral artery disease	1 (25)	1 (2)	-	-

*SAVR*, Surgical aortic valve replacement; *TAVI*, transcatheter aortic valve implantation.
